# Comparative Efficacy of *Botryocladia leptopoda* Extracts in Scar Inhibition and Skin Regeneration: A Study on UV Protection, Collagen Synthesis, and Fibroblast Proliferation

**DOI:** 10.3390/molecules29235688

**Published:** 2024-11-30

**Authors:** Chen-Che Hsieh, Tsung-Kai Yi, Yi-Feng Kao, Shin-Ping Lin, Ming-Chieh Tu, Yu-Chieh Chou, Jheng-Jhe Lu, Huey-Jine Chai, Kuan-Chen Cheng

**Affiliations:** 1 Department of Seafood Science, National Kaohsiung University of Science and Technology, No. 142, Haijhuan Rd., Nanzih District, Kaohsiung 81157, Taiwan; fstcch@nkust.edu.tw; 2 Seafood Technology Division, Fisheries Research Institute, Ministry of Agriculture, Keelung 20246, Taiwan; tkyi@mail.tfrin.gov.tw (T.-K.Y.); yfkao@mail.tfrin.gov.tw (Y.-F.K.); mctu@mail.tfrin.gov.tw (M.-C.T.); 3 School of Food Safety, Taipei Medical University, Taipei 11042, Taiwan; 4 Ph.D. Program in Drug Discovery and Development Industry, College of Pharmacy, Taipei Medical University, 250 Wu-Hsing Street, Taipei 11031, Taiwan; d343111003@tmu.edu.tw; 5 Research Center of Biomedical Device, Taipei Medical University, 250 Wu-Hsing Street, Taipei 11031, Taiwan; 6 TMU Research Center for Digestive Medicine, Taipei Medical University, 250 Wu-Hsing Street, Taipei 11031, Taiwan; 7 Institute of Biotechnology, National Taiwan University, No. 1, Sec. 4, Roosevelt Rd., Taipei 10617, Taiwan; iamlucharlie37@gmail.com; 8 Institute of Food Science and Technology, National Taiwan University, No. 1, Sec. 4, Roosevelt Rd., Taipei 10617, Taiwan; 9 Department of Optometry, Asia University, 500, Lioufeng Rd., Wufeng, Taichung 413, Taiwan; 10 Department of Medical Research, China Medical University Hospital, China Medical University, 91, Hsueh-Shih Road, Taichung 404, Taiwan

**Keywords:** *Botryocladia leptopoda*, collagen synthesis, oxidative stress, fibroblast proliferation, pigmentation modulation

## Abstract

*Botryocladia leptopoda* is a red macroalga known for its bioactive compounds with antioxidant, anti-inflammatory, and skin-regenerative properties. The study aimed to examine their effects on UV protection, collagen synthesis, fibroblast proliferation, and pigmentation modulation. Bioactive compounds were extracted using two solvents, producing ethanol extract (FE) and alkaline extracts (AE). Methods involved characterizing extracts using mass spectrometry and assessing their effects on human fibroblasts under UVB-induced damage. UV absorbance, ROS production, and collagen synthesis were evaluated. The FE extract, which comprised 4-hydroxyquinoline, phytosphingosine, and docosapentaenoic acid, reinstated procollagen type I synthesis to 113% of baseline levels and reduced TGF-β1-mediated fibroblast proliferation to 87.78%. FE also suppressed *Smad2* and *α-SMA* by 71% and 68%, respectively, indicating modulation of fibrosis-associated pathways. AE, containing 4-hydroxyquinoline and phenylalanine betaine, demonstrated dose-responsive cellular repair, reducing fibroblast proliferation to 97.86% and *collagen* Type I expression by 73% at 1000 μg/mL. Both extracts decreased ROS production, with FE and AE reducing levels by 21.4% and 19.7%, respectively, under UVB-induced oxidative stress. FE showed superior scar inhibition, while AE excelled in skin regeneration and pigmentation management.

## 1. Introduction

*Botryocladia leptopoda* is a red alga widely recognized for its significant biosynthesis of bioactive compounds, which are well-documented for their potent antioxidant and anti-inflammatory properties [1]. This alga naturally accumulates phytochemicals under stress conditions such as high light intensity, nutrient deprivation, and saline environments. These phytochemicals have been the subject of numerous studies because they protect the skin from oxidative stress, reduce inflammation, and support wound healing [2,3]. These properties make it a promising ingredient in dermatological applications, particularly in protecting the skin from UV-induced damage and promoting skin regeneration [4]. *B. leptopoda* produces bioactive compounds such as carotenoids, fatty acids, and polyphenols. These compounds contribute to skin health by acting as antioxidants, anti-inflammatory agents, and cell proliferation modulators [1,5].

The beneficial effects of algae, including *B. leptopoda*, in skincare have gained significant attention in recent years due to their natural composition and potential to address various skin conditions. One of the critical challenges in dermatology is managing conditions such as scarring, hyperpigmentation, and fibrosis. The role of bioactive compounds in inhibiting the overproduction of collagen and modulating fibrotic pathways offers the potential for scar treatment and tissue regeneration [6]. Studies have suggested that algae-derived compounds can regulate fibroblast activity, reducing excessive collagen deposition, which is essential for treating hypertrophic scars and fibrosis [7,8]. Furthermore, the capacity of these substances to influence melanogenesis, the biological mechanism underlying pigmentation, positions microalgae as a viable option for interventions aimed at hyperpigmentation conditions, including melasma [9]. In this context, *B. leptopoda* holds potential as a valuable source of bioactive compounds with diverse applications in dermatological therapies.

This investigation explores the therapeutic potential of *B. leptopoda* extracts in dermal care applications, specifically regarding their roles in scar prevention, collagen synthesis augmentation, UV protection, and pigmentation management. By analyzing the impact of *B. leptopoda* extracts on specific cellular pathways integral to skin repair and protection, this study aspires to contribute to the formulation of innovative skincare products that harness the natural advantages of algae.

## 2. Results

### 2.1. Potential Skin Care Compounds in B. leptopoda Extracts

**Figure 1:**
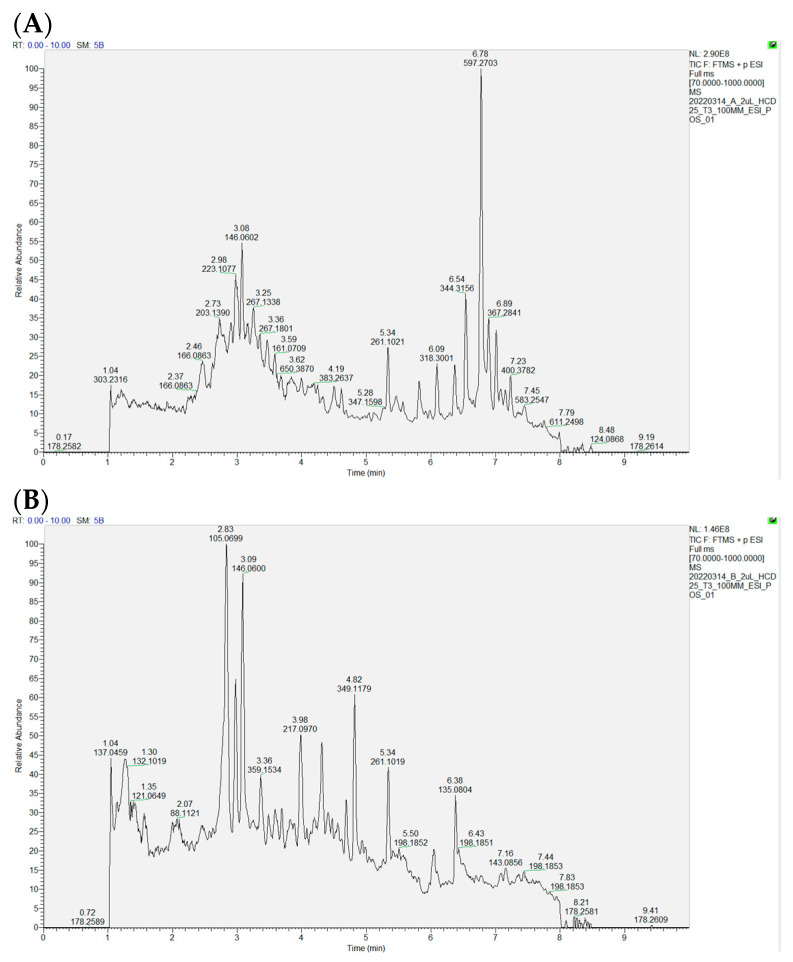
The profile of *B. leptopoda* extracts (FE (**A**) and AE (**B**)). (**A**). FE extraction. (**B**). AE extraction.

Analysis of the ethanol extract (FE) revealed 4-hydroxyquinoline (5.96%), phytosphingosine (3.97%), and docosapentaenoic acid (1.26%). These compounds have been shown to contribute to skin protection, reduce inflammation, and support wound healing [10,11]. On the other hand, the alkaline extract (AE) contained a higher proportion of 4-hydroxyquinoline (9.39%) alongside phenylalanine betaine (5.62%) and γ-aminobutyric acid (1.55%) (Table 1, Figure 1A,B). These substances are recognized for promoting cellular growth, aiding in skin tissue repair, being anti-melanogenic, and regulating immune responses [9,12,13].

The compositional variations observed can plausibly be ascribed to the choice of extraction solvents employed. Ethanol, which can extract polar and nonpolar molecules, permitted the acquisition of a broader spectrum of bioactive constituents within the FE extract. Conversely, sodium hydroxide in the AE extract proficiently compromised the structural integrity of the algal cell walls, thereby enhancing the liberation of specific compounds, including phenylalanine betaine [14]. The solvents utilized during the extraction process were instrumental in determining the bioactive profiles of both the AE and FE extracts, influencing their prospective applications in skin health.

**Table 1:** *B. leptopoda* extracts (FE and AE) composition analysis.

Proportion (%)	Compound	Potential Skin Care Related Function
**FE extraction**		
5.96	4-Hydroxyquinoline	Antioxidant [15,16]
3.97	Phytosphingosine	Skin protection [11]
1.44	1-Methylindole-3-carboxamide	Improve skin barrier [17]
1.26	Docosapentaenoic acid (DPA)	Anti-inflammatory [18]
1.25	Indole-5-carboxylic acid ethyl ester	Immune regulation [12]
**AE extraction**		
9.39	4-Hydroxyquinoline	Antioxidant [15,16]
5.62	Phenylalanine betaine	Anti-melanogenic properties [9]
2.95	Indole-5-carboxylic acid ethyl ester	Immune regulation [12]
1.55	γ-Aminobutyric acid	Skin repair [13]
1.25	1-Methylindole-3-carboxamide	Improve skin barrier [17]

### 2.2. Comparative Analysis of UV Protection, Collagen Synthesis, and Melanin Inhibition Between FE and AE Extracts of B. leptopoda

**Figure 2:**
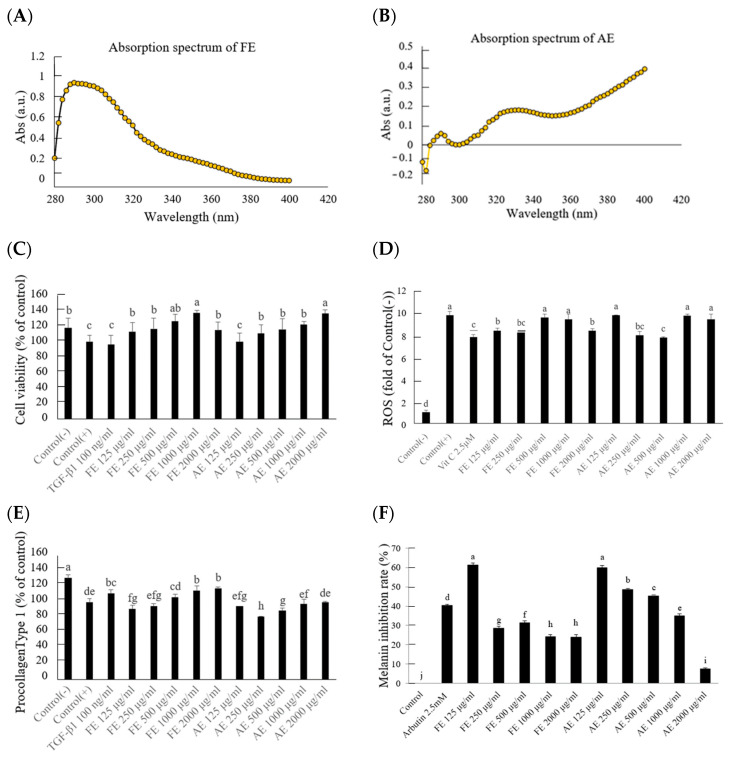
UV protection, collagen synthesis, and melanin inhibition in FE and AE extracts of *B leptopoda*. (**A**) Absorption spectrum of FE extraction; (**B**) Absorption spectrum of AE extraction; (**C**) Cell viability at different doses of treated FE or AE extraction with UVB irradiation; (**D**) Fluorescence intensity changes at different doses of treated FE or AE extraction with UVB irradiation; (**E**) Procollagen Type 1 content changes at different doses of treated FE or AE extraction with UVB irradiation; (**F**) Melanin inhibition rate at different doses of treated FE or AE extraction. The experimental results were repeated three times, and all were expressed in the form of mean ± standard deviation (SD). Different letters indicate significant differences (*p* < 0.05).

The FE extract exhibited strong absorption in the UVB range (280–320 nm), suggesting a significant role in mitigating UVB-induced skin damage (Figure 2A,B). This aligns with 4-hydroxyquinoline, a compound known for its photoprotective and antioxidant capabilities [15,16]. In contrast, the AE extract showed higher absorption in the UVA range (320–400 nm), indicating an enhanced capacity to counteract oxidative stress induced by UVA exposure. The higher levels of phenylalanine betaine and *γ*-aminobutyric acid in AE likely contributed to these protective effects, as these compounds are associated with skin repair and cellular regeneration [13].

Cell viability increased significantly when treated with FE at a 125 μg/mL concentration, where survival rates reached 113% of the UVB-exposed control group (Figure 2C). Statistical analysis indicated significant differences (*p* < 0.05) between the UVB-exposed control group and the treatment groups (FE and AE) at concentrations of 125 μg/mL and higher. This result highlights the extract’s role in facilitating cellular recovery after UVB-induced stress, particularly at optimal concentrations such as 125 μg/mL. The comparative analysis also showed that FE exhibited superior efficacy compared to AE at the same concentration. Previous research on marine algae, including *Dunaliella Salina* and other marine-derived compounds, supports these findings, as algae extracts have consistently shown improvements in cell viability through the reduction in oxidative stress and inflammation caused by UV exposure [19,20]. The antioxidant effects observed in the ROS assay were significant, as demonstrated by reductions of 21.4% and 19.7% in ROS levels with 250 μg/mL FE and AE extracts, respectively, compared to the UVB-exposed group (Figure 2D). Statistical analysis revealed significant differences (*p* < 0.05) between the treated groups (FE and AE) and the UVB-exposed control. The efficacy of FE in reducing ROS levels was marginally greater than that of AE, emphasizing the superior antioxidant potential of FE. However, no statistically significant differences were observed between the FE and AE groups. For instance, previous research on marine algae, *Chlorella vulgaris*, and *Spirulina platensis* has demonstrated similar capacities to lower reactive oxygen species and protect cells from oxidative damage [21]. UVB exposure significantly reduced procollagen type I levels in human fibroblasts [22]. The FE extract, containing compounds like phytosphingosine (3.97%), effectively restored procollagen type I synthesis, achieving a significant increase (*p* < 0.05) at concentrations above 500 μg/mL compared to the UVB-exposed control (Figure 2E). In contrast, the AE extract demonstrated limited efficacy, with no statistically significant improvement observed in collagen synthesis at comparable concentrations. This disparity likely stems from the distinct bioactive profiles of the extracts, where AE’s composition, including phenylalanine betaine, aligns more with anti-melanogenic properties rather than promoting collagen synthesis [9,23,24]. When 125 μg/mL of FE and AE were administered, the melanin inhibition rates were recorded at 61.23% and 59.89%, respectively (Figure 2F). Both extracts demonstrated the ability to limit melanin synthesis and accumulation. Statistical analysis revealed significant differences (*p* < 0.05) between the treated groups (FE and AE) and the UVB-exposed control. For AE, a dose-dependent response was observed at higher concentrations, with inhibition rates improving significantly (*p* < 0.05) at 500 μg/mL compared to 125 μg/mL, indicating that increasing its concentration enhanced melanin inhibition. In contrast, FE exhibited significant melanin inhibition primarily at lower concentrations, such as 125 μg/mL, suggesting that its efficacy might be attributed to the presence of bioactive compounds that exert potent effects even at lower doses. These differences highlight the distinct chemical profiles of FE and AE extracts and their influence on melanin synthesis [9]. This highlights how the chemical differences between FE and AE extracts contribute to their varying efficacy in collagen regulation and anti-melanogenic effects.

### 2.3. Targeted Modulation of Cell Cycle Regulatory Proteins by B. leptopoda Extracts

**Figure 3:**
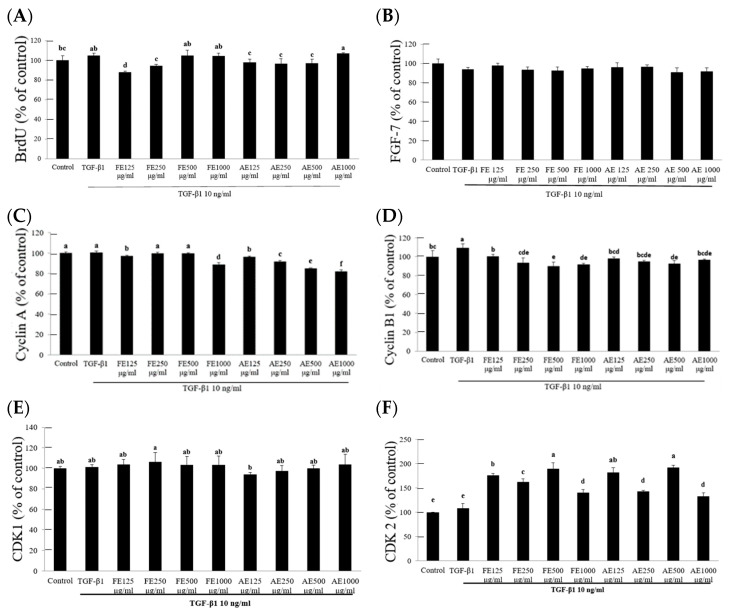
The effects of *B. leptopoda* extracts (FE and AE) on cell cycle regulatory proteins and FGF7 expression in hypertrophic scar fibroblasts. (**A**) BrdU absorbance levels indicate the reduction in TGF-β1-induced cell proliferation after treatment with FE and AE extracts. (**B**) FGF7 expression analysis revealed no significant differences between the experimental and control groups. (**C**,**D**) Inhibition of Cyclin A and B1 expression by FE and AE, demonstrating their regulatory effects on DNA replication and mitosis. (**E**,**F**) Upregulation of CDK2 levels, indicating increased fibroblast entry into the DNA synthesis phase. The experimental results were repeated three times, and all were expressed in the form of mean ± standard deviation (SD). Different letters indicate significant differences (*p* < 0.05).

The BrdU assay indicated that TGF-β1 induced a marked increase in hypertrophic scar fibroblast (HSF) proliferation, with absorbance levels reaching 104.93% relative to the control group. When the *B. leptopoda* extracts were applied at a concentration of 125 μg/mL, proliferation was reduced, with FE lowering the absorbance to 87.78% and AE to 97.86% (Figure 3A). These reductions in cellular proliferation reflect the capacity of both extracts to inhibit TGF-β1-induced growth, which is commonly associated with the development of hypertrophic scars. FE and AE extracts have shown similar effects to those of other algae, like *Spirulina* sp. and *Chlorella* sp., which modulate pathways such as Akt/mTOR to reduce fibroblast proliferation, further supporting the potential of *B. leptopoda* extracts in controlling cell proliferation and inflammation [1,5]. The analysis of FGF7 expression was conducted to assess the effect of FE and AE extracts on hypertrophic scar fibroblast activity. As FGF7 plays a crucial role in wound healing, its expression levels were measured to evaluate any influence from the extracts [25]. The results indicated no significant differences in FGF7 levels between the treatment and control groups, suggesting that neither FE nor AE directly impacted the regulation of FGF7 (Figure 3B).

Cyclins A and B and CDKs regulate cell division and proliferation. Cyclin A is responsible for DNA replication and the transition through the S phase, while Cyclin B regulates mitosis. CDK1 and CDK2 function in tandem with these cyclins to ensure proper cell cycle progression [17,26]. The AE demonstrated a dose-dependent inhibition of Cyclin A, with 250 μg/mL reducing its levels to 91.38% and 1000 μg/mL lowering them to 81.75%, suggesting a significant regulatory effect during the DNA replication phase. FE and AE showed similar inhibitory effects on Cyclin B1, with reductions to 85.53% and 86.76% of control levels at 250 μg/mL, respectively (Figure 3C,D). This indicates the ability to modulate mitotic progression, potentially limiting excessive fibroblast proliferation in hypertrophic scars. However, no significant changes in CDK1 expression were observed, suggesting that the extracts do not directly affect the initiation of the growth phase or mitosis. CDK2, on the other hand, was significantly elevated, reaching 176.30% and 181.58% of control levels with FE and AE, respectively (Figure 3E,F). This upregulation of CDK2 likely accelerated fibroblast entry into the DNA synthesis phase, which is crucial for wound healing [27].

The selective modulation of Cyclin A and B by the *B. leptopoda* extracts, without causing uncontrolled proliferation, aligns with their role in promoting controlled cell growth necessary for wound repair. Unlike extracts from *Spirulina* sp. and *Chlorella* sp., which have shown broader effects on the entire cell cycle [26,28], the extracts from *B. leptopoda* appear to specifically target phases of DNA replication and mitosis, making them potentially safer for long-term therapeutic applications [29].

### 2.4. Comparison of B. leptopoda Extracts in Inhibiting Hypertrophic Scar Contraction

**Figure 4:**
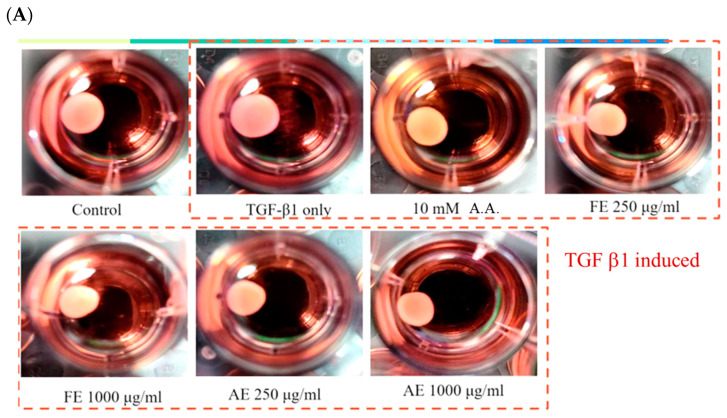

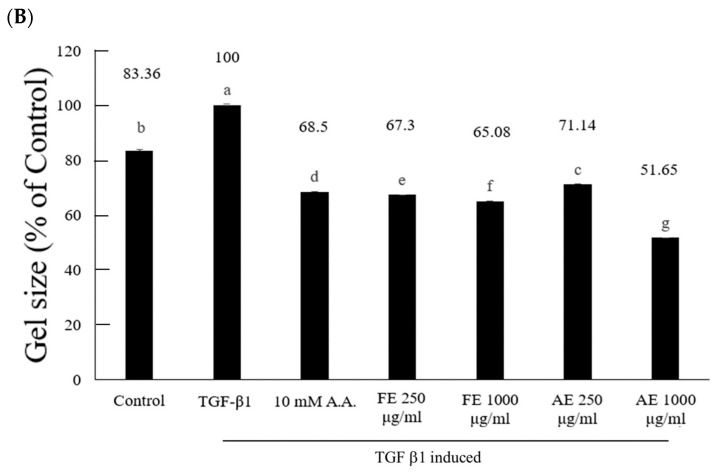
Effect of *B. leptopoda* extracts on hypertrophic scar contraction in human fibroblasts (HSF). (**A**) Images of collagen gels after 48 h of treatment with FE and AE; (**B**) Quantitative analysis of gel contraction. The experimental results were repeated three times, and all were expressed in the form of mean ± standard deviation (SD). Different letters indicate significant differences (*p* < 0.05).

The assessment of *B. leptopoda* extracts (FE and AE) on the contraction of hypertrophic scars in human fibroblasts (HSF) demonstrated that the application of 250 μg/mL FE diminished the scar area to 67.3% of that observed in the control group. This alteration was analogous to the outcome achieved at 68.5% by the commercial anti-scar agent, 6-Acetamidohexanoic acid (A.A.), implying that FE possesses comparable effectiveness in curbing scar proliferation (Figure 4). Such findings corroborate earlier investigations, which indicated that extracts from *B. leptopoda* encompass bioactive compounds such as 4-hydroxyquinoline, phytosphingosine, and docosapentaenoic acid, all of which exhibit anti-inflammatory, antioxidant, and wound-healing characteristics [18,23,30].

In contrast, AE exhibited a marginally lesser effect, reducing 71.14% at 250 μg/mL and 51.65% at 1000 μg/mL, suggesting a dose-responsive relationship. The elevated concentration of phenylalanine betaine and γ-aminobutyric acid (GABA) in AE, both recognized for their roles in enhancing cellular proliferation and tissue repair, may elucidate the variations in effectiveness when juxtaposed with FE [31,32]. While AE demonstrates efficacy, its predominant bioactive constituents appear to be more oriented toward stimulating cellular growth and inhibiting melanogenesis instead of directly impeding fibroblast proliferation, as evidenced by FE. The superior performance of FE can be ascribed to its elevated levels of anti-inflammatory and skin-protective constituents. For example, 4-hydroxyquinoline is acknowledged for its ability to mitigate oxidative stress within fibroblasts, while phytosphingosine and docosapentaenoic acid facilitate skin regeneration and reduction in inflammation [30,32,33]. 

These synergistic effects likely underpin FE’s pronounced inhibitory action on scar contraction. In comparison, while AE presents some efficacy, its composition may render it more appropriate for applications emphasizing tissue repair over scar inhibition.

### 2.5. B. leptopoda Extracts Inhibit TGF-β1-Induced Fibrosis and Collagen Synthesis in Hypertrophic Scar Fibroblasts

**Figure 5:**
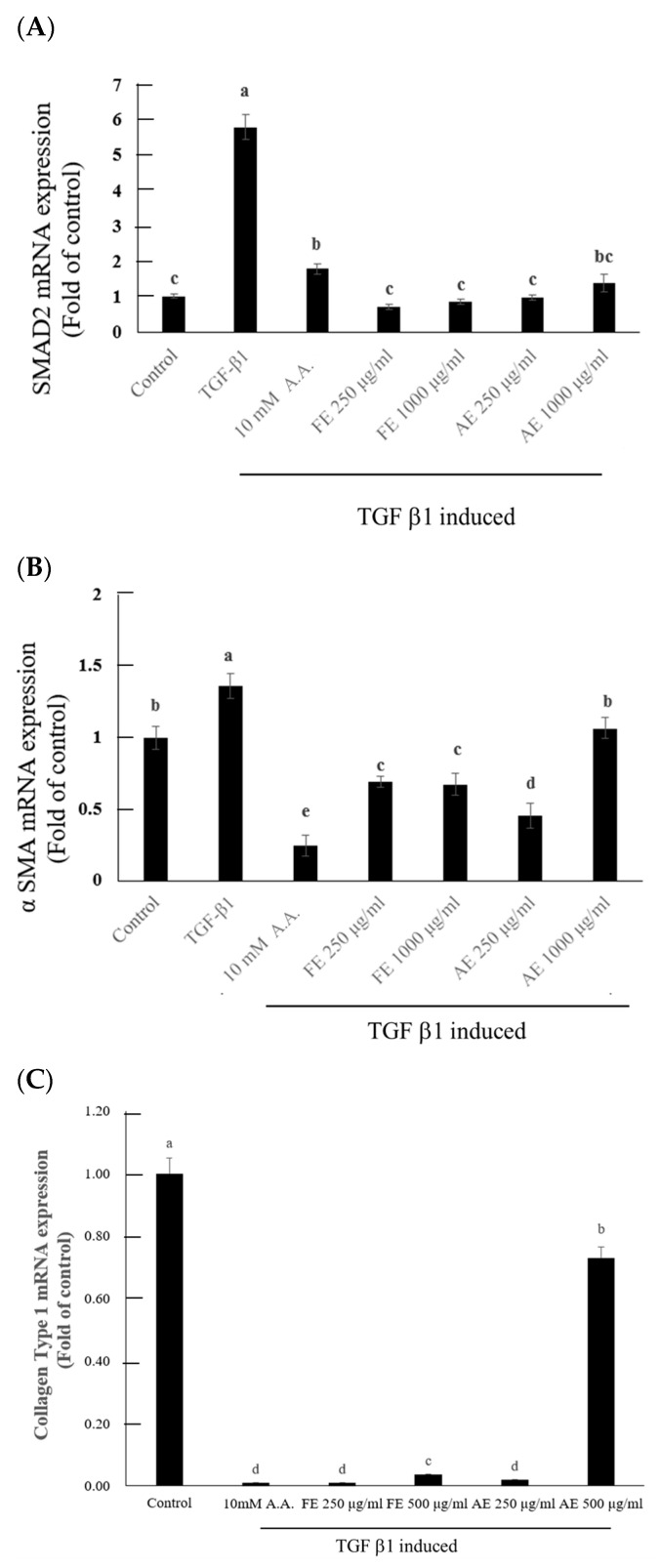
Effects of *B. leptopoda* extracts on TGF-β1-induced fibrosis in hypertrophic scar fibroblasts. (**A**) *Smad2* mRNA expression, (**B**) *α-SMA* mRNA expression, and (**C**) *Collagen Type I* mRNA expression levels. The experimental results were repeated three times, and all were expressed in the form of mean ± standard deviation (SD). Different letters indicate significant differences (*p* < 0.05).

In the investigation of TGF-β receptor suppression in hypertrophic scar fibroblasts, extracts from *B. leptopoda* (FE and AE) were evaluated for their effects on the TGF-β1-induced upregulation of *Smad2* and *α-SMA*, both of which are integral to the progression of fibrosis [34,35,36]. The administration of TGF-β1 resulted in a marked increase in *Smad2* and *α-SMA* mRNA expression levels relative to the control (5.78- and 1.36-fold, respectively). Nonetheless, both FE and AE extracts exhibited a significant downregulation of these markers, especially at a concentration of 250 µg/mL, where Smad2 expression reverted to baseline levels (Figure 5A), indicating a suppressive influence on TGF-β1 signaling pathways. This finding implies that the extracts can proficiently inhibit TGF-β receptor-mediated fibrotic processes, with FE demonstrating slightly superior efficacy to AE in attenuating *Smad2* mRNA levels. Likewise, *α-SMA* expression was notably diminished in the presence of both FE and AE, with reductions to 0.68 and 0.71-fold, respectively, compared to the control (Figure 5B). The findings align with earlier research highlighting the impact of algal phytocompounds in mitigating fibrosis via the modulation of TGF-β signaling pathways [7,8]. The phytochemicals present in FE, such as phytosphingosine and docosapentaenoic acid, likely underpinned its enhanced anti-fibrotic activity [18,23]. In contrast, while demonstrating efficacy, AE seemed to exhibit diminished potency, potentially due to its bioactive composition favoring cellular growth and repair over the inhibition of fibroblast proliferation.

In examining *collagen Type I* mRNA expression, the extracts also revealed varying degrees of effectiveness. The application of FE at 250 µg/mL resulted in a reduction in collagen mRNA expression to undetectable levels, comparable to that of a commercial anti-scar agent (10 mM A.A.) (Figure 5C). This observation suggests that FE is particularly adept at inhibiting collagen biosynthesis, a critical process in scar development [37]. Conversely, AE at elevated concentrations (500 µg/mL) demonstrated partial inhibition of *collagen Type I* expression, indicating that while AE can mitigate scar formation, its efficacy is contingent upon dosage and is somewhat inferior to that of FE. These results imply that FE may represent a more potent anti-fibrotic therapeutic alternative due to its superior capacity to inhibit *Smad2* and *collagen Type I* expression, which are vital pathways in scar formation.

## 3. Materials and Methods

### 3.1. Materials and Chemicals

C57BL/6J mouse murine melanoma (B16F10) and human foreskin fibroblast (Hs68) cell lines were obtained from the American Type Culture Collection (ATCC) (Manassas, VT, USA). Human hypertrophic scar fibroblasts (HSF) cell line HSF106 was obtained from the Cell Research Corporation (Singapore). MTS reagent powder (G1111) and phenazine methosulfate were purchased from GeneLabs Life Science Corp. (Taipei, Taiwan). Procollagen Type IC EIA Kit was purchased from Takara Biomedical Technology (Beijing, China). Smad2/3 colorimetric cell-based ELISA Kit was obtained from Aviva Systems Biology (San Diego, CA, USA). Human α-Smooth muscle actin, α-SMA ELISA Kit was purchased from Cambridge International, LLC (Houston, TX, USA). EDTA solution, trypsin, and antibiotics (penicillin/streptomycin) were obtained from GE Healthcare Life Science (Logan, UT, USA). Vivaspin 15R and Vivaspin Turbo 15 were obtained from Sartorius Stedim Biotech GmbH (Goettingen, Germany). Cell culture media (Dulbecco’s Modified Eagle Medium, or DMEM, supplemented with high glucose, phenol red, sodium pyruvate, and L-glutamine) and fetal bovine serum were retrieved from Hyclone Laboratories Inc. (Logan, UT, USA). α-Melanocyte-stimulating hormone and reactive oxygen species were retrieved from Merck (Burlington, MA, USA). All chemicals used in this study were of analytical grade and purchased from Merck (Burlington, MA, USA).

### 3.2. Preparation of Bioactive Compounds from B. leptopoda

The raw material, *B leptopoda*, was obtained from the Eastern Marine Fishery Research Center of the Ministry of Agriculture (Taitung, Taiwan). The Botryocladia leptopoda specimens used in this study were originally obtained in 2013 from a supplier in Hualien County. The collected algal strains were acclimated and cultivated indoors in 0.5-ton Artemia tanks with natural light and flowing water, employing a suspension culture method to optimize biomass growth. Over a cultivation period of four weeks, the algae demonstrated a growth rate increase of 192%. For long-term preservation, the algal biomass was stored in 9-L fermenter tanks under low-temperature conditions with low biomass density and minimal nutrient supplementation. The culture medium was replaced weekly to ensure the maintenance of viable algal cultures for further experiments. After being thoroughly washed, the algae were dried and pulverized. The *B leptopoda* powder was then extracted at 25 °C using sodium hydroxide (alkaline extract, AE) and ethanol (FE extract), respectively.

### 3.3. Component Identification of B. leptopoda Extracts

Specifically, 10 mg of freeze-dried microalgal biomass was suspended in 1 mL of chromatography-grade methanol with 1% BHT, vortexed for 5 min, and stored at 4 °C for 1h. Subsequently, ultrasonic extraction was performed in an ice bath for 30 min, followed by centrifugation at 12,000× *g*·min^−1^ for 5 min at 4 °C. The supernatant was filtered using 0.22 μm PTFE membranes for further analysis. An ultra-high-performance liquid chromatography (UHPLC) (Thermo Scientific Vanquish Horizon UHPLC System, Milan, Italy) and a quadrupole electrostatic field orbitrap high-resolution mass spectrometer (Q-Orbitrap-HRMS) (Thermo Scientific Vanquish Horizon UHPLC System, Milan, Italy)were employed in this research. The UHPLC conditions included a Syncronis C18 column, specific solvent compositions, a flow rate of 0.3 mL·min^−1^, and an injection volume of 5.0 μL, with gradient elution details referred to by Zhang et al. (2023) [38]. Full MS scan data were collected in positive ion mode at a resolving power of 70,000 FWHM, covering a scanning range of *m*/*z* 100–1200. The automatic gain control target was established at 5 × 10^5^ ions, with a maximum injection duration of 200 ms. Data-dependent acquisition parameters facilitated product ion spectra collection with a collision energy of 45–60 eV, and a resolution power of 35,000 FWHM was implemented. A mass inclusion list, containing precursor ion *m*/*z* of target compounds and acquisition windows, was utilized. The H-ESI source parameters were defined, including gas flow rates, spray voltage, capillary temperature, S lens RF level, and source temperature. In full MS scan mode, precursor ion *m*/*z* values (mass error ≤ 5 ppm) were utilized for quantification, while dd-MS2 modes assured structural confirmation of each compound [38].

### 3.4. Absorption Spectrum Analysis

The quantification of the area beneath the curve for UVA/UVB absorption spectra serves as a methodology for assessing the effectiveness of sun protection. The specimens were subjected to analysis utilizing a UV-Visible spectrophotometer, wherein their absorption spectra were recorded across UVA (320–400 nm) and UVB (280–320 nm) wavelengths. The computed integrated area beneath the curve was employed to ascertain the UV absorption capability [39].

### 3.5. Photodamage Recovery Assay

Cell Viability: Hs 68 human fibroblasts were seeded into 96-well plates and cultured at 37 °C with 5% CO_2_ for 24 h. Following incubation, the cells were treated with a culture medium containing different concentrations of *B. leptopoda* bioactive extracts. The cells were then exposed to UVB radiation for 600 s and subsequently incubated for an additional 24 h. The culture medium was removed, and MTS solution was added. After 4 h, absorbance was measured at 490 nm. Cell viability was calculated by comparing the absorbance values of treated groups to those of the control group, which was set at 100% [40].

Type I Collagen: To assess the impact of UVB radiation on Type I collagen production in Hs 68 human fibroblasts, cells were seeded into 24-well plates at a density of 4 × 10⁴ cells per well, according to the instructions provided in the commercial reagent kit. After cell attachment, the culture medium containing *B. leptopoda* bioactive extracts was added. The cells were cultured for 24 h, after which Type I procollagen was measured using a Procollagen Type I C-Peptide ELISA Kit (TaKaRa MK101, Otsushi, Japan).

UV Damage Prevention Assay: To assess the effect of UVB radiation on reactive oxygen species (ROS) levels in Hs68 human fibroblasts, cells were seeded into 96-well plates at a density of 3 × 10^5^ cells/well. After cell attachment, a medium containing *B. leptopoda* bioactive compounds was added. The cells were then exposed to UVB for 600 s. Following UV exposure, the medium was replaced with a DCFH-DA fluorescent probe solution, and the cells were incubated for 30 min. Fluorescence absorbance was measured to evaluate ROS levels [41].

Melanin Metabolism Assay: The effect of *B. leptopoda* bioactive compounds on melanin production in B16-F10 murine melanocytes (with α-MSH, melanocyte-stimulating hormone) was assessed. B16-F10 cells were seeded into 24-well plates at a density of 5 × 10^4^ cells/well. After cell attachment, a medium containing *B. leptopoda* bioactive compounds (with α-MSH) was added, and the cells were incubated for 48 h. Following incubation, 1N NaOH solution was added, and the plates were heated at 55 °C for 1 h. Absorbance at 405 nm was measured to determine melanin levels [41].

### 3.6. Inhibition Assay for Hypertrophic Fibroblasts

Cell Proliferation Assay: Human hypertrophic scar fibroblasts (HSF) were seeded into 96-well plates at a density of 2 × 10^4^ cells/well. After cell attachment, a medium containing 125–1000 μg/mL of *B. leptopoda* extract was added. A group treated with only 10 ng/mL TGF-β1 served as the positive control, while the untreated group with only culture medium served as the negative control. The fetal bovine serum (FBS) concentration was reduced to 1%. After 48 h of incubation, cell proliferation was measured using the Cell Proliferation ELISA BrdU kit (Merck, Burlington, MA, USA) [42,43].

Fibroblast Growth Factor Expression Analysis: Primary hypertrophic scar fibroblasts derived from the right forearm keloid of a human donor (HSF106, Cell Research Corporation) were seeded into 48-well plates at a density of 4 × 10^4^ cells/well. After cell attachment, the FBS concentration was reduced to 1%, and the cells were treated with a medium containing TGF-β1 and *B. leptopoda* extract. After 24 h of incubation, the supernatant was collected, and FGF levels were measured using an FGF ELISA kit (Lifespan Biosciences, Linwood, WA, USA) [44].

Cell Cycle Analysis: The expression levels of cell cycle regulators, including Cyclin A, Cyclin B, CDK1, and CDK2, were analyzed to assess the inhibitory effect of the extracts on hypertrophic fibroblast proliferation. Human hypertrophic scar fibroblasts (HSF) were seeded into 48-well plates at a density of 4 × 10^4^ cells/well. After cell attachment, the FBS concentration was reduced to 1%, and the cells were treated with a medium containing TGF-β1 and *B. leptopoda* extract [45]. After 24 h of incubation, the supernatant was collected, and the expression levels of human CDK1 and CDK2 ELISA Kit were obtained from Fine Test Biotech Corp., Taipei, Taiwan. Cyclin A and Cyclin B colorimetric cell-based ELISA Kits were obtained from Aviva Systems Biology, San Diego, CA, USA.

### 3.7. Contraction Assay

Human hypertrophic scar fibroblasts (HSF) were mixed with collagen gel from the CytoSelect™ Cell Contraction Assay Kit (Cell Biolabs, Inc., San Diego, CA, USA) and seeded into 24-well plates. After cell attachment, the medium containing *B. leptopoda* extract was added, and cells were induced with 1 ng/mL TGF-β. A commercial drug, 10 mM 6-Acetamidohexanoic acid, was used as a control. After 48 h of incubation, the contracted gel area was measured using ImageJ software 1.50.

### 3.8. Extracellular Matrix Proliferation Assay for Hypertrophic Fibroblasts

TGF-β Receptor Inhibition Assay: The inhibitory effect of the samples on TGF-β receptor function was assessed by analyzing the expression of TGF-β receptor-related factors, *Smad2* (F: 5′-GGCGAATCGGCGGGG-3′; R: 5′-CCTCTTGTATCGAACCTCCCG-3′) and α-SMA (F: 5′-CTGCTGAGCGTGAGATTGTC-3′; R: 5′-CTCAAGGGAGGATGAGGATG-3′), in hypertrophic fibroblasts. Primary fibroblasts from the right forearm keloid of a human donor (HSF106, Cell Research Corporation) were seeded into 48-well plates at a density of 4 × 10^4^ cells/well. After cell attachment, a medium containing TGF-β1 and *B. leptopoda* extract, along with a commercial drug (10 mM 6-Acetamidohexanoic acid), was added. After 48 h of incubation, the gene expression was analyzed by using qPCR analysis [45].

Scar inhibition evaluation of different *B. leptopoda* Extracts: The ability of *B. leptopoda* extracts to inhibit scar formation was evaluated by analyzing collagen I mRNA expression in hypertrophic fibroblasts. Primary fibroblasts from the right forearm keloid of a human donor (HSF106, Cell Research Corporation) were seeded into 48-well plates at a density of 4 × 10^4^ cells/well. After cell attachment, a medium containing *B. leptopoda* extracts and a commercial drug (10 mM 6-Acetamidohexanoic acid) was added. After 48 h of incubation, the expression of the *collagen* Type I (*COL1A1*) (F: 5′-CAGGCAAACCTGGTGAACA-3′; R: 5′-CTCGCCAGGGAAACCTCT-3′) gene fragment was analyzed by using qPCR analysis [10].

RNA samples were reverse transcribed into cDNA by utilizing the CFX Connect Real-time PCR Detection System with 96 × 0.2 mL tubes, 96-well PCR plates, and 12 × 8-tube strips. (Bio-Rad Laboratories, Inc., Hercules, CA, USA) and iScript™ RT-qPCR Sample Preparation Reagent #1708899, iScript™ cDNA Synthesis Kit, 100 × 20 µL rxns #1708891, and iTaq Universal SYBR Green Supermix #172-5124 were purchased from Bio-Rad Laboratories, Inc. (Hercules, CA, USA). Subsequently, quantitative PCR analysis was performed to amplify the promoter region of the DNA fragment. To standardize the results, the relative abundance of housekeeping genes was exploited as the internal standard. Data regarding fold enrichment and the percentage of specific genes were collected and calculated [41].

### 3.9. Statistical Methodology

All experiments were performed in triplicate. The experimental results were expressed as mean ± standard deviation (Mean ± S.D.). Statistical analysis of the data was performed using Duncan’s multiple-range test to assess the differences between groups. A significance level of 0.05 was set for the analysis, which was conducted using the SAS (Statistical Analysis System, Institute Inc., Cary, NC, USA) software, version 6.12 for Windows.

## 4. Conclusions

The research elucidated specific functional characteristics of *B. leptopoda* extracts (FE and AE) about mechanisms pertinent to scar development, dermal protection, and wound healing. The FE extract demonstrated enhanced effectiveness in alleviating UVB-induced harm, diminishing collagen production, and suppressing fibroblast proliferation. Its bioactive constituents, abundant in 4-hydroxyquinoline, phytosphingosine, and docosapentaenoic acid, rendered it particularly advantageous for applications aimed at anti-inflammatory, antioxidant, and skin-regenerative outcomes. These attributes position FE as a formidable candidate for formulations targeting scar mitigation and interventions necessitating robust suppression of fibrotic tissue formation. In contrast, the AE extract, characterized by elevated phenylalanine betaine and γ-aminobutyric acid levels, notably influenced cellular proliferation, tissue repair, and melanogenesis inhibition. Its proficiency in UVA absorption and facilitation of tissue regeneration indicates that AE may be more appropriately employed in contexts emphasizing skin restoration and pigmentation regulation rather than scar mitigation. Nonetheless, AE’s reduced effectiveness in lowering collagen production and fibroblast proliferation relative to FE constrains its applicability for conditions associated with excessive scar tissue formation.

In conclusion, FE seems more aptly suited for clinical strategies focused on scar minimization and controlling fibroblast proliferation. At the same time, AE may be more beneficial for skin repair, pigmentation control, and other regenerative tissue applications. These observations highlight the necessity of customizing the application of *B. leptopoda* extracts according to their bioactive characteristics and the specific dermal health objectives sought. Future investigations could examine the potential synergistic effects of combining both extracts to harness their complementary advantages in dermatological formulations.

## Data Availability

The data presented in this study are available on request from the corresponding author.
